# First-Year Antibiotics Exposure in Relation to Childhood Asthma, Allergies, and Airway Illnesses

**DOI:** 10.3390/ijerph17165700

**Published:** 2020-08-07

**Authors:** Zhijun Zou, Wei Liu, Chen Huang, Chanjuan Sun, Jialing Zhang

**Affiliations:** 1 Department of Building Environment and Energy Engineering, School of Environment and Architecture, University of Shanghai for Science and Technology, Shanghai 200093, China; usstzou@163.com (Z.Z.); huangc@usst.edu.cn (C.H.); sunchanjuan@usst.edu.cn (C.S.); zjialing94@163.com (J.Z.); 2 Institute for Health and Environment, Chongqing University of Science and Technology, Chongqing 401331, China

**Keywords:** antibiotics, asthma, allergies, preschooler, China

## Abstract

*Background*: Associations of early antibiotics exposures with childhood asthma, allergies, and airway illnesses are debated. *Objectives*: We aimed to investigate associations of first-year antibiotics exposure with childhood asthma, allergies, and airway illnesses. *Methods*: A cross-sectional study was conducted among preschoolers in Shanghai, China during 2011–2012. A questionnaire regarding household environment and lifestyles and childhood health outcomes was reported by the child’s parents. *Results*: In total, 13,335 questionnaires (response rate: 85.3%) were analyzed and 3049 (24.1%) children had first-year antibiotics exposure. In the multivariate logistic regression analyses, first-year antibiotics exposure had significant associations with the higher odds of lifetime-ever pneumonia (adjusted OR, 95% CI: 2.15, 1.95–2.37), croup (1.46, 1.24–1.73), wheeze (1.44, 1.30–1.60), asthma (1.38, 1.19–1.61), food allergy (1.29, 1.13–1.46), and allergic rhinitis (1.23, 1.07–1.41), and as well as current (one year before the survey) common cold (≥3 times) (1.38, 1.25–1.52), dry cough (1.27, 1.13–1.42), atopic dermatitis (1.25, 1.09–1.43), wheeze (1.23, 1.10–1.38), and rhinitis symptoms (1.15, 1.04–1.26). These associations were different in children with different individual characteristics (age, sex, family history of atopy, and district) and other early exposures (breastfeeding, home decoration, pet-keeping, and environmental tobacco smoke). *Conclusions*: Our results indicate that first-year antibiotics exposure could be a strong risk factor for childhood pneumonia, asthma, allergies, and their related symptoms. The individual characteristics and other early exposures may modify effects of early antibiotic exposure on childhood allergies and airway illnesses.

## 1. Introduction

Overuse of antibiotics is an urgent issue in worldwide and in China [1,2,3]. Several studies have investigated associations of antibiotics exposures during the early lifetimes with childhood asthma and allergies [1,4,5,6,7,8,9,10,11,12,13,14,15,16,17,18,19,20,21,22,23,24]. However, conclusions among these studies were inconsistent. Several studies suggested that antibiotics exposures during the first years of lifetime were strongly associated with asthma, allergic rhinitis, and atopic dermatitis among preschool children [11,13,14,15,18,24,25]. However, some studies did not support this hypothesis [11,19,26]. Several studies also considered that these strong associations perhaps were confounded by recall bias, indication, and reverse causation [5,10,16]. Besides, two studies reported that the increased risk of asthma for early antibiotics exposures diminished with aging and was not statistically significant after three years of age among children who were exposed to antibiotic in early life [27,28]. A longitudinal follow-up study found that early antibiotic exposures appeared to increase higher risk for atopy among children with <2 than with ≥2 household pets, as well as among children who were breastfed ≥4 than <4 months [14]. However, few studies considered associations of early antibiotics exposure with childhood health outcomes among children with different individual characteristics and early exposures [1,20,29,30,31], particularly in China where prevalences of asthma and allergies have rapidly increased in the past 20 years [32,33,34,35] and where antibiotics have been overused [1,36].

In this study, as a part of the CCHH (China, Children, Homes, Health) study [32,37], we investigated associations of parental-reported antibiotics exposure during the first year of lifetime with nine lifetime-ever allergic and airway diseases/symptoms as well as with five current (within one year before the survey) diseases/symptoms among 4–6-year-old children in Shanghai, China. We also compared these associations in various subgroups. These subgroups were respectively stratified by the potential confounders that included the child’s age, sex, family history of atopy, district, and breastfeeding, as well as home decoration, pet-keeping, environmental tobacco smoke (ETS), and home dampness-related exposures during the first year of the child’s lifetime. We hypothesized that these studied associations could be positive and significant.

## 2. Methods

### 2.1. Studied Population and Questionnaire

We conducted a cross-sectional questionnaire-based study (CCHH phase one) in 72 kindergartens during April 2011–April 2012. These kindergartens were randomly selected from five districts (three urban districts and two suburban districts) of Shanghai city, China. During the teacher–parent meeting in these kindergartens, we surveyed parents/guardians of preschool children by an International Study of Asthma and Allergies in Childhood (ISAAC)-modified questionnaire. There was a total of 15,266 completed and valid questionnaires (response rate: 85.3%). Since age is a key factor in the diagnose of the analyzed diseases [32], we selected 13,335 children 4–6 years old as the studied population and excluded questionnaires for 1–3-year-old (*n* = 782) and 7–8-year-old (*n* = 849) children because sample sizes for these age groups were small. Besides, in each specific analysis, we only selected samples who had full data in all covariates in the analysis, and excluded all samples with any missing data in any covariate in the analysis.

For our questionnaire, questions about the child’s history of asthma, allergies, and airway diseases/symptoms were derived from ISAAC [38]. Questions about dwelling characteristics, household environment, and family lifestyle behaviors for our Chinese version were modified from the Dampness in Building and Health (DBH) study in Sweden [39]. A previous article has provided the full CCHH questionnaire [34]. In our questionnaire, parents/guardians of the studied children provided information regarding first-year antibiotics exposure via the question “Had your child been treated by antibiotics during the first year of the child’s lifetime (yes vs. no)”. Children were classified as having first-year antibiotics exposure when this question was answered “yes”.

All studied health outcomes were reported by the children’s parents. These outcomes included nine lifetime-ever illnesses, namely asthma (doctor-diagnosed), wheeze, croup, pneumonia (doctor-diagnosed), food allergy, allergic rhinitis (doctor-diagnosed), rhinitis symptoms, atopic dermatitis, and ear infections, and five current illnesses, namely wheeze, dry cough, rhinitis symptoms, atopic dermatitis, and common cold (≥3 times). Here, “lifetime-ever” was defined as since birth to the survey day, and “current” was defined as during the past 12 months before the survey. Children were classified as having these outcomes when their questionnaires were answered “yes”. Besides, to further show effects of first-year antibiotics exposure on current health outcomes, we combined lifetime-ever and current illnesses with respect to asthma, wheeze, allergic rhinitis, and rhinitis, and created six combined outcomes (asthma + current wheeze, asthma + no current wheeze, no asthma + current wheeze, allergic rhinitis + current rhinitis, allergic rhinitis + no current rhinitis, and no allergic rhinitis + current rhinitis). In total, 20 health outcomes were studied.

The study was conducted in accordance with the Declaration of Helsinki, and the protocol was approved by the Ethics Committee of School of Public Health, Fudan University (International Registered Number: IRB00002408 & FWA00002399).

### 2.2. Statistical Analyses

According to previous studies [20,37,39,40,41] and our valid data, we classified nine factors, which were supported to be associated with the studied outcomes, as covariates. These factors were the child’s age, sex, family history of atopy, district, duration of breastfeeding, first-year pet-keeping, first-year home dampness-related exposure, first-year home environmental tobacco smoke (ETS), and first-year home decoration. Here, family history of atopy was defined as at least one of the child’s family members having had at least one of the following illnesses: asthma, eczema, and allergic nose or eye problems. First-year home dampness-related exposure was defined as a report of at least one of the following indicators in the home during the first year of the child’s lifetime: visible mold spots and/or damp stains, windowpane condensation, and moldy odor. First-year home ETS was defined as at least one smoker among family members living in the home during the first year of the child’s lifetime. First-year home decoration was defined as that the residence was decorated during the first year of the child’s lifetime.

All statistical analyses were performed by SPSS 19.0 (IBM Corp., Armonk, NY, USA). Pearson’s chi-square test was performed to compare prevalence differences between children with and without first-year antibiotics exposure. Bivariate and multiple logistic regression models were applied to investigate associations between first-year antibiotics exposure and the studied outcomes, with consideration of the covariates mentioned above, among the entire children and among children in various subgroups that were, respectively, stratified using lifetime-ever pneumonia and the above covariates. Associations were indicated by odds ratios (ORs) and adjusted odds ratios (Adjusted ORs) with 95% confidence intervals (CIs). Significances in the statistical analyses were set at *p*-value < 0.05, except in the testing for multiple health outcomes. In the comparisons of prevalences of 20 health outcomes between children with and without first-year antibiotics exposure, their significances were set at the Bonferroni corrected *p*-value (0.05/20 = 0.0025).

## 3. Results

In total, 13,335 (response rate: 85.3%) questionnaires for 4–6-year-old children were collected (Table 1). Boys and girls had similar sample sizes. Sample sizes for children in different ages had a little difference. About one-third of children were from suburban districts. In total, 23.9% of the analyzed children had family history of atopy; 41.8% and 41.4% had breastfeeding >6 months and first-year environmental tobacco smoke, respectively; 12.4% had early home decoration; 12.4% had first-year pet-keeping; and 58.0% had first-year home dampness-related exposures. Overall, 24.1% of the analyzed children had first-year antibiotics exposure. Most of these proportions had significant differences between children with and without first-year antibiotics exposure (Table 1).

Significantly higher prevalences of all studied health outcomes were found in children with first-year antibiotics exposure than in children without first-year antibiotics exposure (*p*-values ≤ 0.001), except for “allergic rhinitis + no current rhinitis” (Table 2).

In the logistic regression analyses among all participants (Table 3), before adjusting the potential confounders, first-year antibiotics exposure was significantly associated with the increased odds of all studied outcomes. After adjusting the potential confounders, first-year antibiotics exposure was still significantly associated with the increased odds of most studied outcomes, including lifetime-ever pneumonia, croup, wheeze, asthma, food allergy, allergic rhinitis, and atopic dermatitis, and current common cold (≥3 times), dry cough, atopic dermatitis, wheeze, and rhinitis symptoms, as well as “asthma + current wheeze” and “allergic rhinitis + current rhinitis”.

In the logistic regression analyses among subgroups that were stratified using lifetime-ever pneumonia (Table 4 and Table 5), more significant associations of first-year antibiotics exposure and the studied outcomes among children without lifetime-ever pneumonia (Table 5) than among children with lifetime-ever pneumonia (Table 4). After adjusting the potential confounders, among children with lifetime-ever pneumonia, only lifetime-ever wheeze and current common cold (≥3 times) were significantly associated with first-year antibiotics exposure. However, among children with lifetime-ever pneumonia, first-year antibiotics exposure was significantly associated with lifetime-ever croup, allergic rhinitis, food allergy, atopic dermatitis, and wheeze; current common cold (≥3 times), atopic dermatitis, dry cough, and rhinitis symptoms; and “asthma+ current wheeze”, “no asthma + current wheeze”, and “allergic rhinitis + current rhinitis”.

Among children in various subgroups which were, respectively, stratified by the child’s individual characteristics and other early exposures (Table 6, Table 7, Table 8 and Table 9), although associations of first-year antibiotics exposure with the studied health outcomes had differences, most associations were positive. For children in different ages, more insignificant associations were found among six-year-old children than among four- and five-year-old children. Boys had stronger associations of first-year antibiotics exposure with current dry cough than girls, whereas girls had stronger associations for lifetime-ever food allergy as well as lifetime-ever and current atopic dermatitis than boys. Compared to children without family history of atopy, children with family history of atopy had weaker associations of first-year antibiotics exposure with lifetime-ever croup, asthma, ear infections, and wheeze as well as current dry cough and wheeze, but had stronger associations for lifetime-ever and current atopic dermatitis. Compared to suburban children, urban children had weaker associations of first-year antibiotics exposure with lifetime-ever croup, asthma, and ear infections, but had stronger associations for lifetime-ever food allergy and current dry cough. Compared to children breastfed ≤6 months, children breastfed >6 months had weaker associations of first-year antibiotics exposure with lifetime-ever asthma, ear infections, allergic rhinitis, food allergy, and pneumonia, but had stronger associations with current atopic dermatitis. Compared to children without first-year home decoration, children with first-year home decoration had stronger associations of first-year antibiotics exposure with lifetime-ever croup, asthma, and pneumonia, as well as current dry cough. Compared to children with first-year pet-keeping, children without pet-keeping had stronger associations of first-year antibiotics exposure with lifetime-ever croup and pneumonia. Compared to children without first-year ETS, children with first-year ETS had stronger associations with lifetime-ever ear infections and pneumonia, as well as current wheeze. Children with first-year home dampness-related exposures only had stronger associations for lifetime-ever allergic rhinitis than children without first-year home dampness-related exposures. Few differences in associations of first-year antibiotics exposure with health outcomes were found between children with and without home dampness-related exposures.

Most of these findings for the individual illnesses were similar to the combinations of lifetime-ever and current illnesses, except among children in different ages (Table 9). We found that four- and five-year-old children had stronger associations of first-year antibiotics exposure with “asthma + current wheeze” and “allergic rhinitis + current rhinitis”, than six-year-old children. No significant associations were found of first-year antibiotics exposure with “allergic rhinitis + no current rhinitis” and “no allergic rhinitis + current rhinitis” in all subgroups (Table 9).

## 4. Discussion

In this cross-sectional questionnaire-based study, we found that first-year antibiotics exposure had significant associations with the increased odds of lifetime-ever pneumonia, croup, wheeze, asthma, food allergy, allergic rhinitis, and atopic dermatitis, as well as current common cold (≥3 times), dry cough, atopic dermatitis, wheeze, and rhinitis symptoms. These associations for most diseases/symptoms were generally stronger among six-year-old children, girls, children without history of atopy, suburban children, children breastfed ≤6 months, children with first-year home decoration, children with first-year ETS exposure, and children without lifetime-ever pneumonia.

Our findings that first-year antibiotics exposure had significant associations with the increased odds of various kinds of allergies and airway illnesses were consistent with the previous studies [4,11,13,15,18,24,42,43,44]. In Phase 3 of the ISAAC study, Foliaki et al. found a significant associations of first-year antibiotic use with current (in the previous 12 months before the survey) wheeze, rhinitis symptoms, and atopic dermatitis among 6–7-year-old children [12]. In a population- and register-based nested case-control study, Metsälä et al. also reported that first-year antibiotics use is associated with an increased risk of childhood asthma diagnosed at age of three years or older [18]. In 2016, Pitter et al. conducted a population-based birth cohort study and reported that children with first-year antibiotic exposure have a higher risk of new-onset asthma [44]. Recently, in two combined population-based cohorts, Ahmadizar et al. found that antibiotic use in early life was associated with an increased risk of asthma [45]. Penders et al. conducted a meta-analysis with 18 longitudinal studies and found that early life antibiotic exposure has significant association with the development of asthma and wheeze [43]. These findings suggested that first-year antibiotics exposure is a strong risk factor for childhood allergies and airway illnesses.

As the previous studies [14,27,28], we found that associations of first-year antibiotics exposure with childhood allergies and airway illnesses could be different among children with different individual characteristics and other early exposures. However, findings in these associations among children in various subgroups were not always consistent between the present study and the previous studies [14,27,28]. In a nationwide population-based study, Ortqvist et al. found that the risk of asthma in children who were exposed to antibiotic in early life diminish with aging and was no longer statistically significant after three years of age among 180,894 children [28]. In a retrospective population-based study in the United States, Ong et al. reported that first-year antibiotic exposure is associated with an increased odd of early-onset asthma which began before three years of age, but has no significant associations with late-onset asthma, which began after three years of age [27]. In the present study, we also found associations of first-year antibiotic exposure with lifetime-ever asthma are statistically significant among four and five-year-old children, but not among six-year-old children. These findings suggested that association of first-year antibiotic exposure with childhood asthma could be confounded by the child’s age and strength of the association may decrease with aging. However, we found that six-year-old children had stronger associations for wheeze than four and five-year-old children. This may be explained that wheeze can only be exactly diagnosed when children are older than six years old [46]. Besides, in a longitudinal follow-up study, Johnson et al. found that early antibiotic exposures appear to increase higher risk for atopy (any positive skin test response for common allergens) among 6–7-year-old children with <2 pets in the home than with ≥2 pets in the home, as well as among children who were breastfed ≥4 months than <4 months [14]. Similar to this study [14], we found that associations of first-year antibiotic exposure with most studied allergies and airway illnesses are weaker among children with than without first-year pet-keeping, but the differences are small. Because sample size for children with first-year pet-keeping was relatively small, findings for these children also could be misled. However, inconsistent with this study [14], we found that children who were breastfed ≤6 months have stronger associations for lifetime-ever asthma, ear infections, allergic rhinitis, food allergy, and pneumonia than children who were breastfed >6 months. Our findings suggest that breastfed >6 months and early pet-keeping may decrease the adverse effects of first-year antibiotics exposure on the childhood health. Furthermore, to our best knowledge, as summarized in the beginning of this section, we also firstly found that associations of first-year antibiotic exposure with certain allergies and airway illnesses are different among children of different sex, as well as under different situations of family history of atopy, early home decoration, and first-year ETS exposure. These findings suggested that association of first-year antibiotic exposure with childhood allergies and airway illnesses could be confounded by individual characteristics and other early exposures. These individual characteristics and other early exposures should be considered in the studies for associations of early antibiotic exposure with childhood allergies and airway illnesses.

Three potential biological mechanisms could support associations between early antibiotics exposure and childhood asthma, allergies, and airway illnesses [10]. First, antibiotics could modify the course of infection as well as reduce the severity and length of infections, thus may lead the child’s immune system towards an allergic pathway. Second, experimental studies have suggested that antibiotics themselves could induce a Th2-skewed response, which may be as an early step in the development of allergies [47]. Third, animal studies have reported that early antibiotic exposure may impact on the gut microbiome and intestinal bacterial diversity, and thus influence the child’s immune system, as well as T helper and regulatory T cell balance [48]. Then, these changes in the child’s immune system increase the susceptibility to the Th2 model of allergies and airway illnesses [49].

Several studies suggested that associations between early antibiotics exposure and the increased risks of childhood asthma, allergies, and airway illnesses could be confounded by recall bias, reverse causation, and indication [4,10,43,44,50]. First, compared to healthy children, recall of early antibiotic exposure could be worse among parents of sick children, or conversely. Such differences could lead spurious associations of early antibiotic use and childhood health in cross-sectional studies using prospectively collected data [10]. Second, reverse causation may occur if children who have early-onset allergic and/or airway diseases/symptoms which were treated with antibiotics. This could be a plausible explanation since wheeze is difficult to be exactly diagnosed in young children [46]. Our finding that lifetime-ever pneumonia has the strongest association with first-year antibiotics exposure seemingly could be partly explained by this reverse causation. Thirdly, Antibiotics were most commonly used to treat respiratory tract infections among infants [10,51]. The respiratory tract infections, particularly those caused by rhinovirus and respiratory syncytial virus (RSV), were suggested to be strongly associated with the increased risk of childhood asthma [42]. Therefore, these infections could be the true reason for the subsequently increased risk of asthma and other allergies, rather than antibiotics that are used to treat these infections [51].

Besides, Wickens and colleagues conducted a cross-sectional study among school children in Steiner (New Zealand) and found that antibiotic use in infancy was associated with childhood asthma and wheeze [50]. However, in a longitudinal birth cohort study, Wickens and colleagues found that association between antibiotic use in infancy and development of asthma in childhood could be confounded by chest infections [51]. This finding is consistent with our finding that associations of antibiotic use in the first year of lifetime with asthma, rhinitis, and airway illnesses were stronger among children without lifetime-ever pneumonia than among children with lifetime-ever pneumonia. The contrasting findings in these studies also suggest that findings in the cross-sectional studies should be treated with caution.

This study had some limitations. First, as with all questionnaire-based cross-sectional studies [43,50], we cannot provide explanations of biological mechanisms for the significant associations of first-year antibiotics exposure and childhood illnesses. Second, as discussed above, all analyzed data were derived from parental-reported questionnaire. Recall bias and misclassification may exist among parents for antibiotics exposure and childhood health information. Thus, our analyzed associations could be confounded. Third, we did not consider the type and dose of antibiotics which were used by the children. Finally, although we adjusted nine potential confounders by the multiple logistic regression analyses, some other potential confounders [20,52], such as genetic factors, family socioeconomic status, ambient air pollution, and early infections, were not considered.

Nevertheless, this study had several strengths. First, the previous studies have confirmed that the ISAAC-based questionnaire is an effective way to obtain the exact information for childhood asthma, allergies, and airway illnesses among children [53]. Second, although some other potential confounders were not considered, we considered nine factors as potential confounders and investigated the target associations among subgroups where were stratified by these factors. Several novel findings were obtained. Third, to our best knowledge, this study is the first study to investigate associations of first-year antibiotics exposure with childhood health outcomes using such large sample sizes in Shanghai, China. Our results that the target associations were much stronger among children without pneumonia than children with pneumonia also further confirmed the adverse effects of first-year antibiotics exposure on childhood asthma, rhinitis, and allergic illnesses. Our findings could provide reference for the guidance of antibiotics use among infants.

## 5. Conclusions

First-year antibiotics exposure is a strong risk factor for childhood asthma, allergies, and airway illnesses. Effects of first-year antibiotics exposure on childhood atopic dermatitis could be stronger on girls and children with family history of atopy. Breastfed longer than six months may decrease adverse effects of first-year antibiotics exposure on childhood health outcomes, whereas first-year home decoration and ETS exposure may enhance these adverse effects.

## Figures and Tables

**Table 1:** Demographic characteristics and covariates (*n* = 13,335).

Items	Sample Sizes, *n* (%)			*p*-Value
		First-Year Antibiotics Exposure		
	Total (*n* = 13,335)	With (*n* = 3049, 24.1%)	Without (*n* = 9618, 75.9%)	
Age (years-old)				
4	5561 (41.7)	1296 (42.5)	4021 (41.8)	
5	4399 (33.0)	1005 (33.0)	3152 (32.8)	
6	3375 (25.3)	748 (24.5)	2445 (25.4)	0.066
Sex				
Boys	6753 (50.6)	1648 (54.0)	4743 (49.3)	
Girls	6536 (49.0)	1393 (45.7)	4845 (50.4)	
Missing data	46 (0.4)	8 (0.3)	30 (0.3)	<0.001
Family history of atopy				
Yes	3097 (23.2)	922 (30.2)	2095 (21.8)	
No	9837 (73.8)	2061 (67.6)	7291 (75.8)	
Missing data	401 (3.0)	66 (2.2)	232 (2.4)	<0.001
District				
Urban	9014 (67.6)	2030 (66.6)	6569 (68.3)	
Suburban	4062 (30.5)	970 (31.8)	2886 (30.0)	
Missing data	259 (1.9)	49 (1.6)	163 (1.7)	<0.001
Duration of breastfeeding				
>6 months	5456 (40.9)	1147 (37.6)	3998 (41.6)	
≤6 months	7605 (57.0)	1857 (60.9)	5431 (56.5)	
Missing data	274 (2.1)	45 (1.5)	189 (2.0)	<0.001
Early home decoration				
Yes	4381 (32.9)	1087 (35.7)	3120 (32.4)	
No	7396 (55.4)	1659 (54.4)	5425 (56.4)	
Missing data	1558 (11.7)	303 (9.9)	1073 (11.2)	<0.001
First-year pet-keeping				
Yes	1628 (12.2)	385 (12.6)	1157 (12.0)	
No	11515 (86.4)	2627 (86.2)	8339 (86.7)	
Missing data	192 (1.4)	37 (1.2)	122 (1.3)	<0.001
First-year environmental tobacco smoke				
Yes	5444 (40.8)	1225 (40.2)	3917 (40.7)	
No	7713 (57.8)	1790 (58.7)	5590 (58.1)	
Missing data	178 (1.3)	34 (1.1)	111 (1.2)	<0.001
First-year home dampness-related exposures				
Yes	6905 (51.8)	1757 (57.6)	4912 (51.0)	
No	5006 (37.5)	967 (31.7)	3805 (39.6)	
Missing data	1424 (10.7)	325 (10.7)	901 (9.4)	<0.001

**Table 2:** Comparisons of prevalences for childhood asthma, allergies, and airway illnesses among children with different situation of antibiotics exposure in the first year of lifetime (Yes vs. No).

Illness	Prevalence, *n* (%)		*p*-Value
	Yes	No	
Lifetime-ever			
Pneumonia	1431 (48.2)	2768 (29.7)	<0.001
Croup	309 (10.6)	619 (6.7)	<0.001
Wheeze	1082 (35.9)	2502 (26.3)	<0.001
Asthma	412 (13.9)	873 (9.3)	<0.001
Food allergy	560 (23.3)	1378 (17.8)	<0.001
Allergic rhinitis	479 (16.2)	1100 (11.7)	<0.001
Atopic dermatitis	772 (26.9)	1980 (21.8)	<0.001
Ear infections	399 (13.3)	996 (10.5)	<0.001
Rhinitis symptoms	1716 (57.2)	5066 (53.5)	<0.001
Current (one year before the survey)			
Common cold (≥3 times)	1527 (51.6)	3839 (41.6)	<0.001
Dry cough	720 (23.8)	1782 (18.7)	<0.001
Atopic dermatitis	490 (16.6)	1163 (12.4)	<0.001
Wheeze	787 (26.1)	1964 (20.6)	<0.001
Rhinitis symptoms	1427 (47.9)	3919 (41.8)	<0.001
Lifetime-ever + Current			
Asthma + current wheeze	284 (12.0)	569 (7.4)	<0.001
Asthma + no current wheeze	122 (5.6)	290 (3.9)	0.001
No asthma + current wheeze	473 (18.6)	1330 (15.7)	0.001
Allergic rhinitis + current rhinitis	399 (21.7)	874 (14.5)	<0.001
Allergic rhinitis + no current rhinitis	77 (5.1)	204 (3.8)	0.029
No allergic rhinitis + current rhinitis	989 (40.8)	2942 (36.4)	<0.001

**Table 3:** Associations between first-year antibiotics exposure and childhood asthma, allergies, and airway illnesses.

Illness	OR, 95% CI (*p*-Value)	Adjusted OR ^a^, 95% CI (*p*-Value)
Lifetime-ever		
Pneumonia	2.21, 2.03–2.41 (<0.001)	2.15, 1.95–2.37 (<0.001)
Croup	1.65, 1.43–1.91 (<0.001)	1.46, 1.24–1.73 (<0.001)
Wheeze	1.57, 1.44–1.71 (<0.001)	1.44, 1.30–1.60 (<0.001)
Asthma	1.57, 1.39–1.78 (<0.001)	1.38, 1.19–1.61 (<0.001)
Food allergy	1.41, 1.26–1.57 (<0.001)	1.29, 1.13–1.46 (<0.001)
Allergic rhinitis	1.45, 1.29–1.63 (<0.001)	1.23, 1.07–1.41 (0.004)
Atopic dermatitis	1.32, 1.20–1.45 (<0.001)	1.17, 1.05–1.31 (0.005)
Ear infections	1.31, 1.15–1.48 (<0.001)	1.15, 1.00–1.33 (0.050)
Rhinitis symptoms	1.16, 1.07–1.26 (<0.001)	1.06, 0.97–1.17 (0.212)
Current (one year before the survey)		
Common cold (≥3 times)	1.50, 1.38–1.63 (<0.001)	1.38, 1.25–1.52 (<0.001)
Dry cough	1.36, 1.23–1.50 (<0.001)	1.27, 1.13–1.42 (<0.001)
Atopic dermatitis	1.40, 1.25–1.57 (<0.001)	1.25, 1.09–1.43 (0.001)
Wheeze	1.36, 1.23–1.49 (<0.001)	1.23, 1.10–1.38 (<0.001)
Rhinitis symptoms	1.28, 1.18–1.39 (<0.001)	1.15, 1.04–1.26 (0.007)
Lifetime-ever + Current		
Asthma + current wheeze ^b^	1.72, 1.48–2.00 (<0.001)	1.50, 1.25–1.79 (<0.001)
Asthma + no current wheeze ^b^	1.45, 1.17–1.80 (0.001)	1.23, 0.95–1.58 (0.120)
No asthma + current wheeze ^b^	1.22, 1.09–1.37 (0.001)	1.14, 0.99–1.30 (0.062)
Allergic rhinitis + current rhinitis ^c^	1.63, 1.43–1.86 (<0.001)	1.34, 1.14–1.58 (<0.001)
Allergic rhinitis + no current rhinitis ^c^	1.35, 1.03–1.77 (0.029)	1.06, 0.77–1.46 (0.735)
No allergic rhinitis + current rhinitis ^c^	1.20, 1.10–1.32 (<0.001)	1.10, 0.99–1.22 (0.090)

^a^ Adjusted for the child’s age, sex, district, family history of atopy, breastfeeding, early home decoration, first-year pet-keeping, first-year environmental tobacco smoke (ETS), and first-year home dampness-related exposures. ^b^ The reference group was children with no asthma and no current wheeze. ^c^ The reference group was children with no allergic rhinitis and no current rhinitis symptoms.

**Table 4:** Associations between first-year antibiotics exposure and childhood asthma, allergies, and airway illnesses, among children with lifetime-ever pneumonia.

Illness	OR, 95% CI (*p*-Value)	Adjusted OR ^a^, 95% CI (*p*-Value)
Lifetime-ever		
Croup	1.27, 1.04–1.54 (0.017)	0.82, 0.14–4.77 (0.823)
Asthma	1.14, 0.97–1.40 (0.110)	1.03, 0.82–1.28 (0.815)
Ear infections	1.08, 0.90–1.30 (0.422)	0.95, 0.75–1.20 (0.661)
Allergic rhinitis	1.12, 0.95–1.32 (0.171)	1.01, 0.81–1.25 (0.961)
Food allergy	1.20, 1.02–1.44 (0.033)	1.12, 0.90–1.39 (0.311)
Atopic dermatitis	1.12, 0.97–1.30 (0.120)	1.05, 0.87–1.26 (0.641)
Wheeze	1.23, 1.08–1.40 (0.002)	1.20, 1.01–1.42 (0.037)
Rhinitis symptoms	1.02, 0.90–1.17 (0.733)	1.05, 0.88–1.24 (0.615)
Current (one year before the survey)		
Common cold (≥3 times)	1.31, 1.15–1.49 (<0.001)	1.20, 1.02–1.42 (0.031)
Atopic dermatitis	1.23, 1.04–1.46 (0.018)	1.10, 0.88–1.38 (0.390)
Dry cough	1.07, 0.93–1.24 (0.335)	1.08, 0.90–1.29 (0.432)
Wheeze	1.06, 0.92–1.23 (0.388)	1.04, 0.86–1.25 (0.689)
Rhinitis symptoms	1.18, 1.04–1.35 (0.010)	1.17, 0.99–1.38 (0.071)
Lifetime-ever + Current		
Asthma + current wheeze ^b^	1.20, 0.98–1.46 (0.077)	1.10, 0.87–1.40 (0.417)
Asthma + no current wheeze ^b^	1.05, 0.80–1.38 (0.719)	0.91, 0.66–1.25 (0.545)
No asthma + current wheeze ^b^	0.97, 0.81–1.17 (0.768)	0.95, 0.77–1.16 (0.601)
Allergic rhinitis + current rhinitis ^c^	1.24, 1.03–1.50 (0.024)	1.03, 0.81–1.31 (0.803)
Allergic rhinitis + no current rhinitis ^c^	1.11, 0.76–1.62 (0.579)	0.90, 0.58–1.40 (0.647)
No allergic rhinitis + current rhinitis ^c^	1.18, 1.02–1.37 (0.024)	1.14, 0.96–1.35 (0.137)

^a^ Adjusted for the child’s age, sex, district, family history of atopy, breastfeeding, early home decoration, first-year pet-keeping, first-year environmental tobacco smoke (ETS), and first-year home dampness-related exposures. ^b^ The reference group was children with no asthma and no current wheeze. ^c^ The reference group was children with no allergic rhinitis and no current rhinitis symptoms.

**Table 5:** Associations between first-year antibiotics exposure and childhood asthma, allergies, and airway illnesses, among children without lifetime-ever pneumonia.

Illness	OR, 95% CI (*p*-Value)	Adjusted OR ^a^, 95% CI (*p*-Value)
Lifetime-ever		
Croup	1.59, 1.26–2.00 (<0.001)	1.38, 1.04–1.84 (0.027)
Asthma	1.49, 1.21–1.85 (<0.001)	1.31, 0.99–1.73 (0.057)
Ear infections	1.34, 1.13–1.60 (0.001)	1.24, 0.99–1.55 (0.058)
Allergic rhinitis	1.46, 1.22–1.74 (<0.001)	1.42, 1.13–1.78 (0.003)
Food allergy	1.46, 1.24–1.68 (<0.001)	1.42, 1.18–1.72 (<0.001)
Atopic dermatitis	1.37, 1.20–1.57 (<0.001)	1.30, 1.10–1.54 (0.003)
Wheeze	1.51, 1.33–1.71 (<0.001)	1.46, 1.24–1.71 (<0.001)
Rhinitis symptoms	1.07, 0.96–1.20 (0.211)	0.99, 0.86–1.15 (0.932)
Current (one year before the survey)		
Common cold (≥3 times)	1.43, 1.21–1.68 (<0.001)	1.42, 1.16–1.74 (0.001)
Atopic dermatitis	2.15, 1.23–3.78 (0.008)	1.21, 1.01–1.45 (0.036)
Dry cough	1.40, 1.22–1.61 (<0.001)	1.33, 1.12–1.59 (0.001)
Wheeze	1.14, 1.02–1.28 (0.021)	1.07, 0.92–1.23 (0.399)
Rhinitis symptoms	1.48, 1.32–1.66 (<0.001)	1.40, 1.22–1.62 (<0.001)
Lifetime-ever + Current		
Asthma + current wheeze ^b^	1.72, 1.34–2.21 (<0.001)	1.57, 1.17–2.10 (0.003)
Asthma + no current wheeze ^b^	1.22, 0.83–1.80 (0.316)	1.07, 0.67–1.70 (0.788)
No asthma + current wheeze ^b^	1.31, 1.12–1.53 (0.001)	1.22, 1.01–1.46 (0.036)
Allergic rhinitis + current rhinitis ^c^	1.58, 1.30–1.93 (<0.001)	1.37, 1.08–1.74 (0.011)
Allergic rhinitis + no current rhinitis ^c^	1.23, 0.82–1.85 (0.325)	0.88, 0.53–1.46 (0.614)
No allergic rhinitis + current rhinitis ^c^	1.06, 0.93–1.20 (0.376)	0.95, 0.82–1.10 (0.482)

^a^ Adjusted for the child’s age, sex, district, family history of atopy, breastfeeding, early home decoration, first-year pet-keeping, first-year environmental tobacco smoke (ETS), and first-year home dampness-related exposures. ^b^ The reference group was children with no asthma and no current wheeze. ^c^ The reference group was children with no allergic rhinitis and no current rhinitis symptoms.

**Table 6:** Associations of first-year antibiotics exposure with asthma, wheeze, and dry cough in subgroups.

Items	AOR ^a^, 95% CI (*p*-Value)				
	Lifetime Asthma	Lifetime Wheeze	Lifetime Croup	Current Wheeze	Current Dry Cough
Sex					
Boys	1.39, 1.15–1.69 (0.001)	1.43, 1.24–1.64 (<0.001)	1.49, 1.19–1.85 (<0.001)	1.20, 0.98–1.46 (0.073)	1.39, 1.15–1.69 (0.001)
Girls	1.37, 1.08–1.73 (0.009)	1.47, 1.26–1.71 (<0.001)	1.43, 1.11–1.84 (0.006)	1.18, 0.99–1.40 (0.060)	1.09, 0.92–1.28 (0.337)
Age (year-old)					
4	1.44, 1.10–1.89 (0.008)	1.40, 1.17–1.66 (<0.001)	1.22, 0.91–1.63 (0.185)	1.25, 1.04–1.51 (0.019)	1.50, 1.25–1.79 (<0.001)
5	1.38, 1.04–1.83 (0.028)	1.39, 1.14–1.69 (0.001)	1.78, 1.31–2.40 (<0.001)	1.17, 0.94–1.44 (0.163)	1.17, 0.94–1.45 (0.169)
6	1.14, 0.81–1.59 (0.453)	1.72, 1.36–2.17 (<0.001)	1.37, 0.91–2.06 (0.135)	1.39, 1.08–1.80 (0.012)	0.94, 0.70–1.26 (0.668)
Family history of atopy					
Yes	1.24, 1.01–1.54 (0.049)	1.36, 1.14–1.62 (0.001)	1.19, 0.92–1.54 (0.183)	1.12, 0.93–1.35 (0.234)	0.84, 0.26–2.75 (0.771)
No	1.53, 1.25–1.88 (<0.001)	1.49, 1.31–1.69 (<0.001)	1.71, 1.38–2.23 (<0.001)	1.30, 1.13–1.50 (<0.001)	1.32, 1.14–1.52 (<0.001)
District					
Urban	1.32, 1.11–1.57 (0.002)	1.46, 1.29–1.65 (<0.001)	1.37, 1.12–1.67 (0.002)	1.24, 1.09–1.43 (0.002)	1.33, 1.16–1.52 (<0.001)
Suburban	1.55, 1.15–2.09 (0.004)	1.39, 1.16–1.67 (<0.001)	1.68, 1.24–2.28 (0.001)	1.18, 0.97–1.44 (0.091)	1.11, 0.90–1.38 (0.332)
Duration of breastfeeding					
>6 months	1.11, 0.85–1.45 (0.430)	1.50, 1.32–1.70 (<0.001)	1.50, 1.22–1.85 (<0.001)	1.50, 1.32–1.70 (<0.001)	1.29, 1.07–1.56 (0.008)
≤6 months	1.54, 1.28–1.85 (<0.001)	1.35, 1.14–1.60 (<0.001)	1.39, 1.06–1.84 (0.019)	1.35, 1.14–1.60 (<0.001)	1.26, 1.09–1.45 (0.001)
Early home decoration					
Yes	1.55, 1.23–1.95 (<0.001)	1.48, 1.25–1.74 (<0.001)	1.61, 1.24–2.09 (<0.001)	1.27, 1.06–1.51 (0.009)	1.40, 1.18–1.67 (<0.001)
No	1.27, 1.04–1.54 (0.018)	1.41, 1.24–1.61 (<0.001)	1.36, 1.10–1.69 (0.005)	1.20, 1.04–1.39 (0.013)	1.18, 1.02–1.37 (0.028)
First-year pet-keeping					
Yes	1.38, 0.93–2.07 (0.113)	1.49, 1.11–1.99 (0.007)	0.95, 0.59–1.51 (0.813)	1.49, 1.11–1.99 (0.007)	1.14, 0.83–1.56 (0.432)
No	1.39, 1.18–1.63 (<0.001)	1.43, 1.28–1.60 (<0.001)	1.56, 1.30–1.86 (<0.001)	1.43, 1.28–1.60 (<0.001)	1.29, 1.14–1.45 (<0.001)
First-year environmental tobacco smoke					
Yes	1.32, 1.04–1.67 (0.023)	1.50, 1.28–1.76 (<0.001)	1.49, 1.15–1.92 (0.002)	1.33, 1.12-1.58 (0.001)	1.24, 1.04–1.49 (0.019)
No	1.43, 1.18–1.74 (<0.001)	1.40, 1.23–1.60 (<0.001)	1.45, 1.16–1.80 (0.001)	1.17, 1.01-1.35 (0.038)	1.29, 1.11–1.49 (0.001)
First-year home dampness-related exposures					
Yes	1.37, 1.14–1.64 (0.001)	1.42, 1.25–1.61 (<0.001)	1.41, 1.16–1.72 (0.001)	1.22, 1.07-1.40 (0.004)	1.25, 1.09–1.44 (0.001)
No	1.39, 1.07–1.81 (0.013)	1.47, 1.23–1.75 (<0.001)	1.55, 1.13–2.12 (0.007)	1.23, 1.01-1.50 (0.040)	1.28, 1.05–1.56 (0.013)

^a^ The adjusted factors for various subgroups were all factors in Table 2 for the entire children except factor for the stratification.

**Table 7:** Associations of first-year antibiotics exposure with pneumonia, allergic rhinitis, and rhinitis symptoms in subgroups.

Items	AOR ^a^, 95% CI (*p*-Value)			
	Lifetime Pneumonia	Lifetime Allergic Rhinitis	Lifetime Rhinitis Symptoms	Current Rhinitis Symptoms
Sex				
Boys	2.18, 1.91–2.49 (<0.001)	1.22, 1.02–1.47 (0.030)	1.00, 0.87–1.14 (0.981)	1.22, 1.02–1.47 (0.030)
Girls	2.12, 1.84–2.45 (<0.001)	1.24, 1.00–1.54 (0.049)	1.14, 0.99–1.32 (0.060)	1.18, 1.03–1.36 (0.022)
Age (year-old)				
4	1.82, 1.55–2.14 (<0.001)	1.42, 1.11–1.80 (0.005)	1.02, 0.87–1.19 (0.857)	1.10, 0.94–1.30 (0.237)
5	2.26, 1.87–2.72 (<0.001)	1.29, 1.01–1.67 (0.049)	1.34, 1.11–1.62 (0.002)	1.43, 1.19–1.72 (<0.001)
6	2.36, 1.90–2.95 (<0.001)	1.20, 0.87–1.65 (0.276)	0.94, 0.76–1.17 (0.602)	1.06, 0.85–1.32 (0.628)
Family history of atopy				
Yes	2.14, 1.79–2.56 (<0.001)	1.24, 1.02–1.49 (0.029)	1.14, 0.94–1.38 (0.188)	1.21, 1.01–1.46 (0.039)
No	2.16, 1.92–2.42 (<0.001)	1.22, 1.00–1.50 (0.049)	1.05, 0.94–1.17 (0.430)	1.12, 1.01–1.26 (0.048)
District				
Urban	2.14, 1.91–2.40 (<0.001)	1.21, 1.03–1.41 (0.020)	1.06, 0.94–1.19 (0.375)	1.15, 1.02–1.29 (0.020)
Suburban	2.18, 1.83–2.61 (<0.001)	1.30, 0.97–1.73 (0.079)	1.10, 0.93–1.31 (0.273)	1.16, 0.97–1.38 (0.108)
Duration of breastfeeding				
>6 months	2.05, 1.75–2.40 (<0.001)	1.10, 0.86–1.41 (0.448)	1.05, 0.90–1.23 (0.521)	1.12, 0.95–1.31 (0.169)
≤6 months	2.21, 1.96–2.50 (<0.001)	1.30, 1.10–1.54 (0.002)	1.07, 0.94–1.21 (0.295)	1.16, 1.02–1.31 (0.019)
Early home decoration				
Yes	2.38, 2.03–2.79 (<0.001)	1.15, 0.93–1.43 (0.195)	1.08, 0.92–1.26 (0.354)	1.10, 0.94–1.28 (0.256)
No	2.02, 1.78–2.28 (<0.001)	1.29, 1.07–1.54 (0.006)	1.05, 0.93–1.19 (0.407)	1.18, 1.04–1.33 (0.010)
First-year pet-keeping				
Yes	1.84, 1.40–2.43 (<0.001)	1.37, 0.95–1.98 (0.089)	1.06, 0.81–1.40 (0.669)	1.12, 0.86–1.48 (0.401)
No	2.19, 1.98–2.43 (<0.001)	1.21, 1.04–1.41 (0.012)	1.07, 0.96–1.18 (0.226)	1.15, 1.04–1.28 (0.009)
First-year environmental tobacco smoke				
Yes	2.25, 1.93–2.63 (<0.001)	1.25, 1.01–1.56 (0.048)	1.11, 0.95–1.29 (0.195)	1.17, 1.00–1.36 (0.049)
No	2.08, 1.84–2.36 (<0.001)	1.22, 1.02–1.46 (0.029)	1.03, 0.91–1.17 (0.604)	1.13, 1.00–1.28 (0.049)
First-year home dampness-related exposures				
Yes	2.14, 1.90–2.42 (<0.001)	1.29, 1.09–1.52 (0.003)	1.07, 0.95–1.21 (0.276)	1.18, 1.04–1.33 (0.009)
No	2.18, 1.86–2.56 (<0.001)	1.10, 0.86–1.41 (0.459)	1.04, 0.89–1.22 (0.610)	1.17, 0.83–1.64 (0.379)

^a^ The adjusted factors for various subgroups were all factors in Table 2 for the entire children except factor for the stratification.

**Table 8:** Associations of first-year antibiotics exposure with ear infections, food allergy, atopic dermatitis, and common cold in subgroups.

Items	AOR ^a^, 95% CI (*p*-Value)				
	Lifetime Ear Infections	Lifetime Food Allergy	Lifetime Atopic Dermatitis	Current Atopic Dermatitis	Current Common Cold (≥3 Times)
Sex					
Boys	1.20, 0.98–1.46 (0.073)	1.15, 0.97–1.38 (0.112)	1.10, 0.94–1.28 (0.244)	1.49, 1.19–1.85 (<0.001)	1.15, 0.97–1.38 (0.112)
Girls	1.11, 0.90–1.37 (0.332)	1.45, 1.21–1.75 (<0.001)	1.26, 1.07–1.48 (0.005)	1.35, 1.12–1.64 (0.002)	1.37, 1.19–1.58 (<0.001)
Age (year-old)					
4	1.02, 0.79–1.33 (0.859)	1.45, 1.18–1.79 (<0.001)	1.21, 1.01–1.45 (0.047)	1.35, 1.09–1.68 (0.005)	1.53, 1.31–1.80 (<0.001)
5	1.07, 0.81–1.40 (0.644)	1.20, 0.93–1.54 (0.163)	1.29, 1.04–1.61 (0.020)	1.23, 0.95–1.60 (0.124)	1.24, 1.03–1.48 (0.023)
6	1.50, 1.10–2.04 (0.010)	1.18, 0.88–1.60 (0.271)	1.10, 0.84–1.44 (0.485)	1.28, 0.93–1.77 (0.125)	1.37, 1.10–1.70 (0.005)
Family history of atopy					
Yes	1.03, 0.81–1.31 (0.834)	1.22, 0.99–1.50 (0.067)	1.35, 1.13–1.62 (0.001)	1.38, 1.13–1.69 (0.002)	1.48, 1.24–1.77 (<0.001)
No	1.23, 1.03–1.47 (0.024)	1.33, 1.14–1.57 (<0.001)	1.08, 0.93–1.24 (0.318)	1.16, 0.97–1.39 (0.097)	1.34, 1.20–1.50 (<0.001)
District					
Urban	1.08, 0.91–1.28 (0.397)	1.33, 1.14–1.54 (<0.001)	1.11, 0.89–1.39 (0.360)	1.22, 1.05–1.42 (0.011)	1.34, 1.19–1.50 (<0.001)
Suburban	1.34, 1.03–1.74 (0.032)	1.16, 0.91–1.49 (0.228)	1.19, 1.05–1.36 (0.009)	1.32, 1.01–1.72 (0.042)	1.46, 1.23–1.73 (<0.001)
Duration of breastfeeding					
>6 months	1.12, 0.88–1.42 (0.351)	1.16, 0.93–1.44 (0.183)	1.21, 1.01–1.46 (0.042)	1.42, 1.14–1.76 (0.001)	1.40, 1.20–1.64 (<0.001)
≤6 months	1.18, 0.98–1.41 (0.081)	1.37, 1.17–1.61 (<0.001)	1.15, 1.00–1.33 (0.049)	1.15, 0.98–1.37 (0.095)	1.37, 1.21–1.54 (<0.001)
Early home decoration					
Yes	1.13, 0.90–1.41 (0.297)	1.31, 1.08–1.60 (0.008)	1.11, 0.93–1.32 (0.256)	1.22, 0.76–1.95 (0.409)	1.42, 1.22–1.66 (<0.001)
No	1.18, 0.98–1.42 (0.084)	1.26, 1.07–1.50 (0.006)	1.22, 1.06–1.42 (0.007)	1.34, 1.13–1.59 (0.001)	1.35, 1.20–1.53 (<0.001)
First-year pet-keeping					
Yes	1.08, 0.72–1.60 (0.722)	1.20, 0.84–1.71 (0.326)	1.18, 0.87–1.61 (0.297)	1.20, 0.84–1.72 (0.320)	1.34, 1.02–1.77 (0.036)
No	1.17, 1.01–1.36 (0.047)	1.30, 1.13–1.49 (<0.001)	1.17, 1.04–1.32 (0.011)	1.26, 1.09–1.45 (0.002)	1.38, 1.25–1.53 (<0.001)
First-year environmental tobacco smoke					
Yes	1.36, 1.09–1.71 (0.007)	1.28, 1.03–1.58 (0.023)	1.18, 0.99–1.42 (0.072)	1.30, 1.05–1.61 (0.017)	1.35, 1.16–1.58 (<0.001)
No	1.04, 0.86–1.25 (0.697)	1.29, 1.10–1.52 (0.002)	1.17, 1.01–1.35 (0.036)	1.22, 1.03–1.44 (0.024)	1.39, 1.23–1.58 (<0.001)
First-year home dampness-related exposures					
Yes	1.16, 0.98–1.38 (0.094)	1.29, 1.10–1.51 (0.002)	1.18, 1.02–1.35 (0.022)	1.22, 1.04–1.44 (0.016)	1.42, 1.26–1.60 (<0.001)
No	1.16, 0.98–1.38 (0.094)	1.29, 1.03–1.61 (0.025)	1.17, 0.96–1.42 (0.111)	1.32, 1.05–1.66 (0.019)	1.29, 1.10–1.51 (0.002)

^a^ The adjusted factors for various subgroups were all factors in Table 2 for the entire children except factor for the stratification.

**Table 9:** Associations of first-year antibiotics exposure with the combinations of lifetime-ever and current illnesses in subgroups.

Items	AOR ^a^, 95% CI (*p*-Value)					
	Asthma + Current Wheeze ^b^	Asthma + No Current Wheeze ^b^	No Asthma + Current Wheeze ^b^	Allergic Rhinitis + Current Rhinitis ^c^	Allergic Rhinitis + No Current Rhinitis ^c^	No Allergic Rhinitis + Current Rhinitis ^c^
Sex						
Boys	1.62, 1.12–2.35 (0.011)	1.08, 0.57–2.02 (0.821)	1.21, 0.94–1.54 (0.134)	1.39, 1.01–1.91 (0.044)	0.93, 0.46–1.87 (0.830)	0.96, 0.78–1.20 (0.964)
Girls	1.44, 0.89–2.33 (0.137)	1.05, 0.52–2.12 (0.894)	1.24, 0.94–1.63 (0.124)	1.36, 0.94–1.97 (0.106)	0.86, 0.41–1.82 (0.697)	0.94, 0.77–1.15 (0.556)
Age (year-old)						
4	1.65, 1.04–2.61 (0.031)	1.20, 0.56–2.57 (0.632)	1.19, 0.91–1.55 (0.197)	1.56, 1.09–2.24 (0.016)	0.84, 0.38–1.86 (0.668)	0.79, 0.63–1.08 (0.061)
5	2.12, 1.34–3.36 (0.001)	1.50, 0.72–3.16 (0.281)	1.51, 1.04–2.19 (0.032)	1.56, 1.02–2.37 (0.039)	1.51, 0.69–3.29 (0.299)	1.29, 0.99–1.68 (0.058)
6	0.72, 0.34–1.54 (0.400)	0.51, 0.17–1.51 (0.226)	1.07, 0.75–1.50 (0.720)	0.93, 0.55–1.58 (0.789)	0.35, 0.08–1.53 (0.164)	0.93, 0.68–1.27 (0.636)
Family history of atopy						
Yes	1.26, 0.82–1.93 (0.288)	0.99, 0.49–1.98 (0.974)	1.09, 0.79–1.51 (0.608)	1.50, 1.07–2.10 (0.020)	0.97, 0.45–2.11 (0.944)	1.07, 0.78–1.45 (0.682)
No	1.97, 1.32–2.94 (0.001)	1.19, 0.63–2.25 (0.598)	1.29, 1.03–1.60 (0.025)	1.28, 0.90–1.81 (0.173)	0.84, 0.42–1.66 (0.606)	0.93, 0.78–1.10 (0.396)
District						
Urban	1.41, 0.99–2.00 (0.053)	1.08, 0.63–1.82 (0.789)	1.24, 0.99–1.57 (0.067)	1.42, 1.08–1.88 (0.013)	0.73, 0.38–1.41 (0.347)	0.95, 0.80–1.14 (0.588)
Suburban	2.02, 1.16–3.52 (0.012)	0.97, 0.34–2.71 (0.947)	1.20, 0.90–1.62 (0.220)	1.27, 0.76–2.12 (0.360)	1.30, 0.56–3.05 (0.539)	0.99, 0.76–1.29 (0.938)
Duration of breastfeeding						
>6 months	0.98, 0.57–1.71 (0.953)	0.95, 0.43–2.07 (0.890)	1.07, 0.83–1.37 (0.607)	1.10, 0.72–1.68 (0.669)	0.52, 0.26–1.07 (0.076)	0.96, 0.79–1.16 (0.680)
≤6 months	1.94, 1.36–2.75 (<0.001)	1.15, 0.64–2.07 (0.645)	1.43, 1.09–1.87 (0.010)	1.56, 1.16–2.10 (0.004)	2.00, 0.92–4.34 (0.078)	0.94, 0.74–1.19 (0.591)
Early home decoration						
Yes	1.33, 0.90–1.95 (0.149)	0.93, 0.43–2.04 (0.859)	1.03, 0.75–1.40 (0.873)	1.26, 0.85–1.89 (0.255)	0.65, 0.27–1.56 (0.333)	0.99, 0.82–1.19 (0.889)
No	2.04, 1.28–3.23 (0.003)	1.17, 0.66–2.10 (0.612)	1.34, 1.07–1.68 (0.011)	1.43, 1.06–1.94 (0.021)	1.03, 0.55–1.93 (0.935)	0.88, 0.69–1.13 (0.317)
First-year pet-keeping						
Yes	1.41, 0.66–2.99 (0.372)	1.02, 0.28–3.80 (0.972)	1.21, 0.74–1.99 (0.456)	1.30, 0.99–1.69 (0.054)	0.24, 0.03–1.85 (0.169)	0.98, 0.64–1.48 (0.908)
No	1.60, 1.17–2.21 (0.004)	1.10, 0.66–1.81 (0.719)	1.22, 1.01–1.48 (0.049)	1.89, 1.03–3.49 (0.040)	1.03, 0.61–1.76 (0.905)	0.95, 0.81–1.11 (0.497)
First-year environmental tobacco smoke						
Yes	1.35, 0.84–2.18 (0.211)	1.01, 0.48–2.10 (0.994)	1.28, 0.96–1.70 (0.090)	1.27, 0.85–1.90 (0.248)	1.05, 0.47–2.32 (0.905)	0.92, 0.72–1.16 (0.469)
No	1.71, 1.18–2.49 (0.005)	1.14, 0.62–2.10 (0.670)	1.18, 0.93–1.50 (0.177)	1.43, 1.06–1.94 (0.020)	0.80, 0.41–1.57 (0.523)	0.97, 0.81–1.17 (0.774)
First-year home dampness-related exposures						
Yes	1.63, 1.14–2.32 (0.007)	1.34, 0.76–2.35 (0.317)	1.27, 1.02–1.58 (0.034)	1.64, 1.08–2.49 (0.020)	0.96, 0.51–1.80 (0.895)	0.93, 0.77–1.12 (0.452)
No	1.33, 0.79–2.24 (0.278)	0.70, 0.29–1.68 (0.424)	1.11, 0.80–1.54 (0.538)	1.24, 0.92–1.67 (0.153)	0.83, 0.34–2.01 (0.673)	0.96, 0.75–1.23 (0.766)

^a^ The adjusted factors for various subgroups were all factors in Table 2 for the entire children except factor for the stratification. ^b^ The reference group was children with no asthma and no current wheeze. ^c^ The reference group was children with no allergic rhinitis and no current rhinitis symptoms.
